# A case of coexistent poorly differentiated adenosquamous carcinoma (glassy cell carcinoma), usual-type adenocarcinoma, and squamous cell carcinoma in situ of the cervix

**DOI:** 10.1007/s00795-023-00354-z

**Published:** 2023-05-02

**Authors:** Kouki Habara, Asami Nishikori, Jin Kiyama, Manami Nakashima, Masanori Koda, Kenji Sasaki, Tomohisa Sakashita, Norifumi Tanaka, Shuji Yonehara

**Affiliations:** 1 Department of Pathology, Japan Agricultural Cooperatives Onomichi General Hospital, 1-10-23 Hirahara, Onomichi, Hiroshima, Japan; 2 Department of Molecular Hematopathology, Faculty of Health Sciences, Okayama University School of Medicine, 2-5-1 Shikata, Kita-Ku, Okayama, Japan; 3 Department of Medical Technology, Okayama University Hospital, 2-5-1 Shikata, Kita-ku, Okayama, Japan; 4 Department of Obstetrics and Gynecology, Japan Agricultural Cooperatives Onomichi General Hospital, 1-10-23 Hirahara, Onomichi, Hiroshima, Japan; 5 Department of Obstetrics and Gynecology, National Hospital Organization East Hiroshima Medical Center, 513 Jike, Saijo, Higashihiroshima, Hiroshima, Japan

**Keywords:** Glassy cell carcinoma, Usual-type adenocarcinoma, Squamous cell carcinoma in situ, Fragment analysis, Human papillomavirus, Loss of heterozygosity

## Abstract

Poorly differentiated adenosquamous carcinoma (glassy cell carcinoma) of the cervix is extremely rare, accounting for 1–2% of all cervical cancers. Herein, we report a case with coexistent poorly differentiated adenosquamous carcinoma (glassy cell carcinoma), “usual-type” adenocarcinoma, and squamous cell carcinoma in situ of the cervix. A female patient in her 60 s was referred to our hospital and diagnosed with poorly differentiated adenosquamous carcinoma based on cervical cytology and biopsy. The tumor was classified as clinical stage IB1 cervical cancer following magnetic resonance imaging; radical hysterectomy was performed. Histopathological examination revealed poorly differentiated adenosquamous carcinoma (glassy cell carcinoma), usual-type adenocarcinoma, and squamous cell carcinoma in situ, all coexisting. All carcinoma regions showed identical sizes to high-risk human papillomavirus (HPV) in fragment analysis. The patient is currently alive, without evidence of recurrence, 31 months post surgery. In this case, three different carcinomas coexisted. Fragment analysis of the patient’s HPV status suggested that all carcinomas were related to an infection with the same high-risk HPV type. To determine the precise mechanism of tumor development, i.e., whether the tumors were of the mixed or collision type, further studies are needed, including clonal analysis for the loss of heterozygosity pattern.

## Introduction

Poorly differentiated adenosquamous carcinoma (previously categorized as glassy cell carcinoma) of the cervix is rare, accounting for only 1–2% of all cervical cancers [1]. It rarely occurs in the presence of other histological malignant tumors in the cervix [2]. Compared with other cervical cancers, poorly differentiated adenosquamous carcinoma of the cervix predominantly occurs between the ages of 31 and 41 years, with a tendency to occur in younger patients [1]. In many cases, it is clinically detected as advanced cancer. It is generally associated with a poor prognosis because it is often resistant to radiation therapy and prone to metastasis [3]. Glassy cell carcinoma was first described by Cherry and Gluckmann [4] in 1956 as a specific and distinct entity of the most poorly differentiated adenosquamous carcinomas. Histologically, glassy cell carcinoma is characterized by abundant cells, ground-glass cytoplasm, distinct cell borders, and high-grade nuclei with prominent nucleoli. Two decades later, Littman et al. [5] performed an in-depth evaluation of glassy cell carcinoma, which resulted in the redefinition and expansion of the histological criteria. Accordingly, glassy cell carcinoma was defined in the 4th edition of the World Health Organization classification as a subtype of “other epithelial tumors” [6]; meanwhile, the most recent 5th edition of the World Health Organization classification discourages the use of “glassy cell carcinoma” and has deallocated it as a specific histological subtype [7]. Therefore, herein, glassy cell carcinoma is described as “poorly differentiated adenosquamous carcinoma (glassy cell carcinoma).”

Immunological evidence has shown that the oncogenic process of cervical cancer involves human papillomavirus (HPV) [8]. Moreover, glassy cell carcinoma is strongly associated with high-risk HPV [9], while adenocarcinoma and squamous cell carcinoma are strongly associated with HPV infection [10]. A review of the literature revealed that cases of glassy cell carcinoma coexisting with other malignant tumors are extremely rare, and only three cases have been reported in the past [11–13]. No cases of coexisting tumors and HPV infection status have been reported thus far. We report a rare case of coexisting poorly differentiated adenosquamous carcinoma (glassy cell carcinoma), usual-type adenocarcinoma, and squamous cell carcinoma in situ of the cervix. Additionally, based on the results of fragment analysis of HPV isolated from the patient, we clarified the relationship between these three tumors and HPV infection and, based on previous studies, discussed the clonality of coexisting tumors.

## Case presentation

**Fig. 1:**
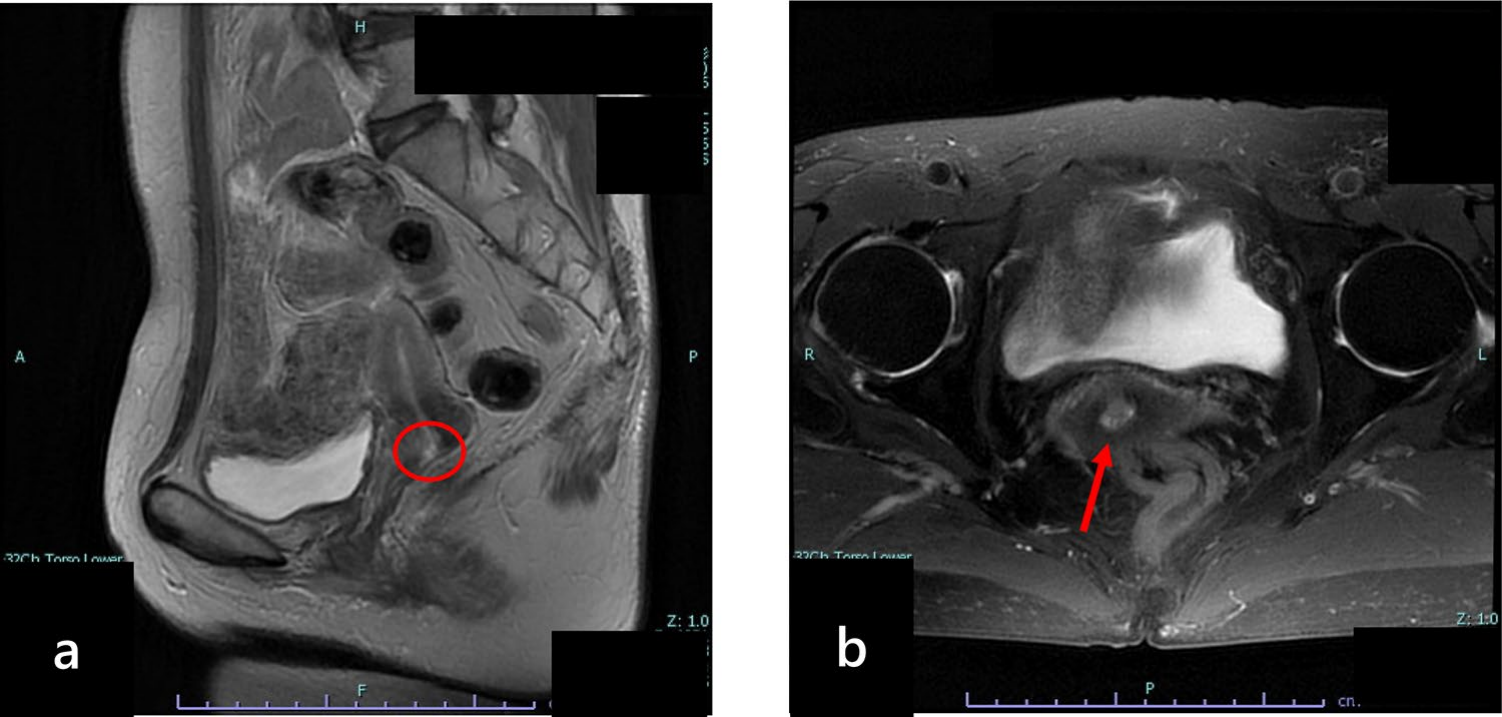
MRI of the pelvis. A faint high-signal intensity area measuring 7 mm in size is visible in the anterior wall of the cervix on T2-weighted MRI. MRI: magnetic resonance imaging. (**a**, longitudinal image; **b**, cross-sectional image)

The patient, a female in her 60 s, had been referred to another hospital 33 months prior with a complaint of hematuria. Physical examination revealed no obvious abnormal findings. She was referred to our hospital’s Department of Obstetrics and Gynecology (Japan Agricultural Cooperatives Onomichi General Hospital, Hiroshima, Japan) for detailed examination and treatment. Pelvic and colposcopic examinations at the initial visit revealed a polyp-like lesion exposed from the inside of the cervical canal. T2-weighted magnetic resonance imaging (MRI) revealed a faint high-signal intensity area measuring 7 mm in size on the anterior wall of the cervix (Fig. 1). Glassy cell carcinoma was suspected based on the cervical cytology findings. The patient was diagnosed with glassy cell carcinoma after a biopsy (before the 5th edition of the World Health Organization classification was published). The tumor was confined to the cervix and classified as clinical stage IB1 cervical cancer. An extensive hysterectomy was performed. Chemotherapy and radiotherapy were not performed at the request of the patient. The patient is currently alive, without evidence of recurrence, 31 months following the surgery.

### Cytological findings

**Fig. 2:**
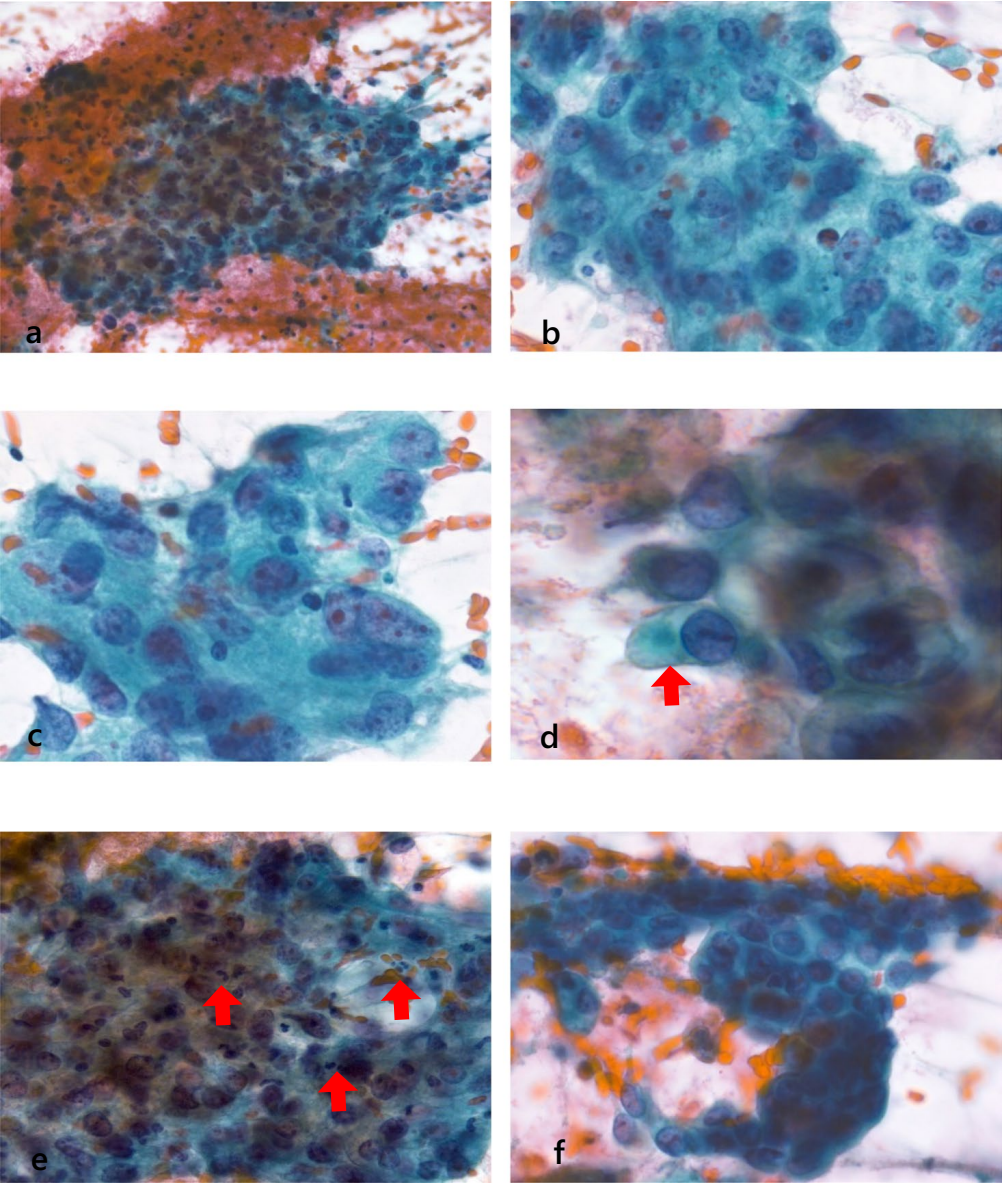
Cytological findings of the cervix. (**a**) Mildly overlapping cell clusters are visible on a hemorrhagic, necrotic, and inflammatory backgrounds (Papanicolaou staining, × 20). (**b, c**) The clusters are solid with no obvious formation of glandular or stratified structures. The boundaries of the cells are relatively unclear and contain dense, fine-to-coarse granular cytoplasm. The nuclei are round to elliptical, with poor irregularity, and centrally located. Some nuclei are eccentrically located. Increased amounts of fine-to-coarse granular chromatin are observed, with one or a few prominent nucleoli (Papanicolaou staining, × 63). (**d**) Cells with mucus-like substance (red arrow) in the cytoplasm are visible (Papanicolaou staining, × 63). (**e**) Inflammatory cell infiltrates, mainly consisting of neutrophils (red arrows), in the cell clusters (Papanicolaou staining, × 40). (**f**) Well-differentiated adenocarcinoma cell clusters forming papillary or tubular structures (Papanicolaou staining, × 63)

Cytological specimens showed many cell clusters in hemorrhagic, necrotic, and inflammatory backgrounds (Fig. 2a). The clusters were solid, with no evidence of a glandular or stratified structure. The cellular boundaries were slightly unclear, with a moderate amount of cytoplasm that stained faintly or densely in light green. Moreover, the nuclei were round to elliptical, with one or a few prominent nucleoli. Most nuclei were central, but some were eccentrically located (Fig. 2b, c). A few cells contained mucus-like substances in their cytoplasm (Fig. 2d). Based on these cytological findings, we suspected glassy cell carcinoma. In addition, these cells were mixed with inflammatory infiltrates composed mainly of neutrophils (Fig. 2e). A review of the cytological specimens revealed a small number of well-differentiated adenocarcinoma cell clusters with the formation of papillary or tubular structures (Fig. 2f). No atypical squamous cells suggestive of carcinoma in situ were observed.

### Histopathological findings

**Fig. 3:**
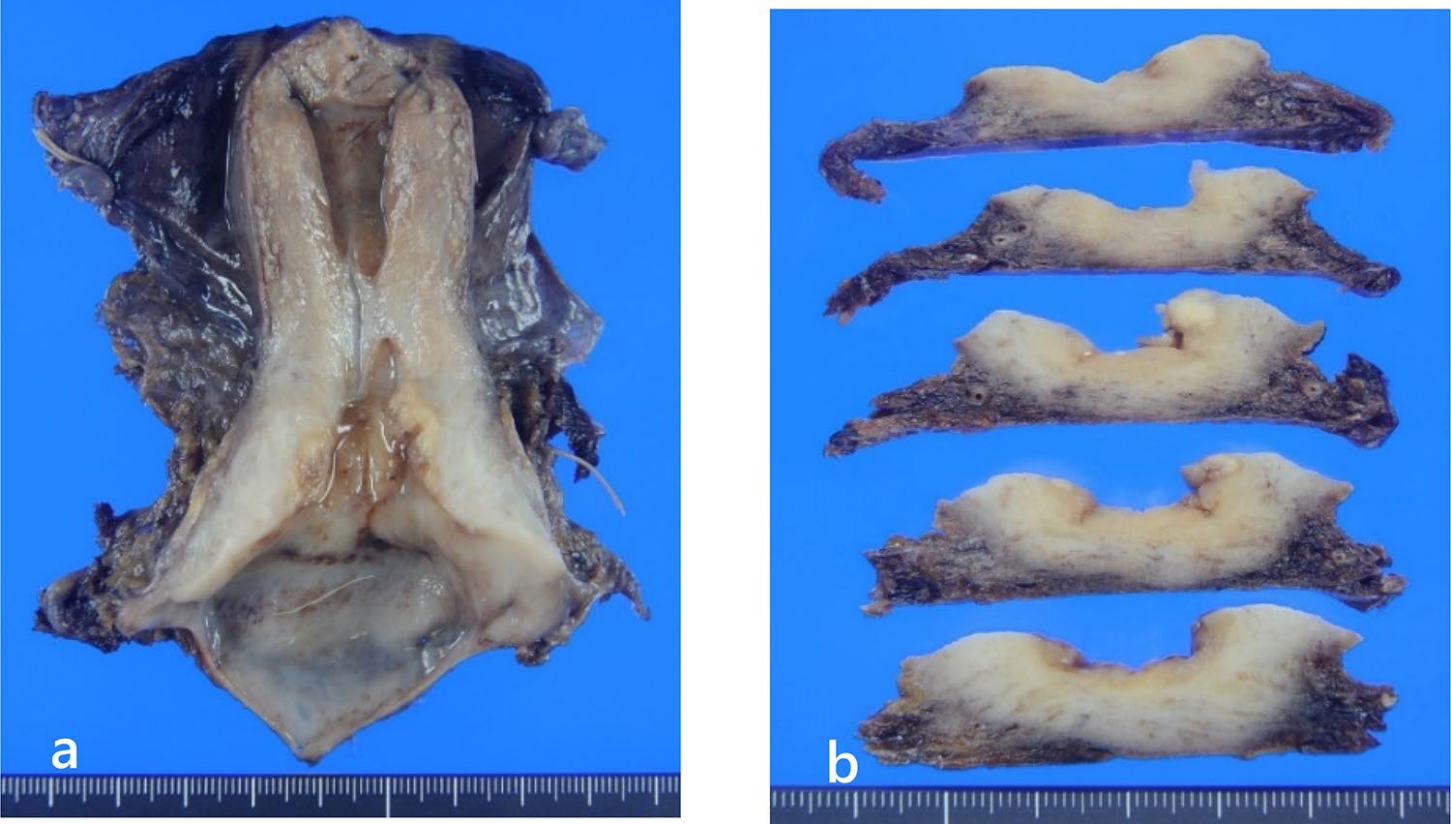
A gray-white solid tumor measuring 8 × 5 × 4 mm in the cervix (**a**: macroscopic image; **b**: cut surface image)

**Fig. 4:**
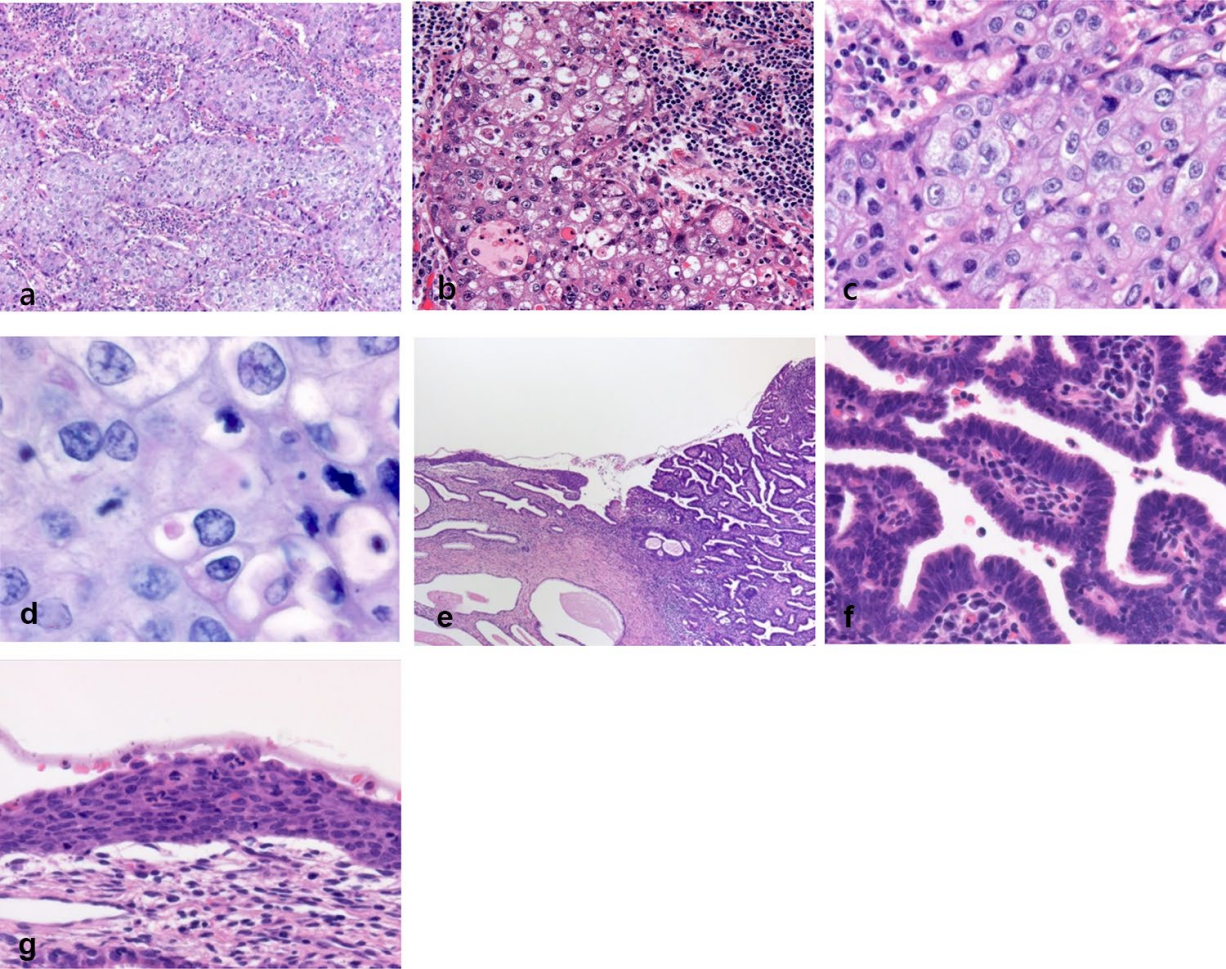
Histopathological examination of the cervix. (**a**) Tumor cell proliferation is observed in the form of solid nests, and intense inflammatory cell infiltration, mainly of plasma cells, is observed in the stroma (hematoxylin and eosin staining, × 10). (**b**) Inflammatory cell infiltration of tumor nests (hematoxylin and eosin staining, × 20). (**c**) Boundaries of the tumor cells forming the nests are clear, with the cells showing abundant glassy acidophilic cytoplasm and round nuclei with large nucleoli (hematoxylin and eosin staining, × 40). (**d**) Histopathological findings in the cervix (mucicarmine staining). A small number of drop-like substances are visible in the tumor cell cytoplasm, exhibiting positive staining for mucicarmine, representing epithelial mucus (mucicarmine staining, × 63) (**e**) Poorly differentiated adenosquamous carcinoma (glassy cell carcinoma), usual-type adenocarcinoma, and squamous cell carcinoma in situ components (hematoxylin and eosin staining, × 4). (**f**) Tall columnar tumor cells showing dense proliferation and formation of small irregular tubules (hematoxylin and eosin staining, × 40). (**g**) Atypical cells showing nuclear swelling and a marked increase in nuclear density proliferating in all layers of the epithelium. No invasion beyond the basement membrane (hematoxylin and eosin staining; × 40)

The macroscopic appearance of the resected uterus is shown in Fig. 3. The total tumor size was 8  × 5  × 4 mm. The tumor grew rapidly, and the stroma showed high infiltration of inflammatory cells, mainly plasma cells (Fig. 4a). Inflammatory cell infiltration of some tumor nests was also observed (Fig. 4b). The boundaries of the tumor cells were relatively clear. The eosinophilic cytoplasm resembled ground glass with round nuclei containing prominent nucleoli (Fig. 4c). Moreover, a small number of drop-like substances were observed in the tumor cell cytoplasm. These were positive for mucicarmine staining. Therefore, they were considered to be epithelial mucus (Fig. 4d). Based on these histological findings, we diagnosed the patient with glassy cell carcinoma. In addition to poorly differentiated adenosquamous carcinoma (glassy cell carcinoma), usual-type adenocarcinoma and squamous cell carcinoma in situ were found in close proximity (Fig. 4e–g). However, there were no areas where the three carcinomas were clearly in contact or transition areas where they overlapped. P16 immunohistochemical staining was strongly positive in all three carcinomas.

### Polymerase chain reaction (PCR) and HPV fragment analysis

PCR was performed using the sample solution (ThinPrep) used for liquid-based cytology of the cervical cytology specimens. It showed that the patient was HPV-18-positive. Next, we prepared tissue samples for hematoxylin and eosin staining from the formalin-fixed paraffin-embedded sections and confirmed the location of each carcinoma. Afterward, tumor cells of each histological type were scraped from the unstained tissue specimens and subjected to DNA extraction. The QIAamp DNA Micro Kit (Qiagen, Valencia, CA, USA) was used for DNA extraction. PCR was then performed using HPV E6 and E7 sequences. PCR was performed using primers that could amplify the high-risk (16, 18, 31, 33, 35, 52b, and 58) and low-risk (6 and 11) types of HPV-DNA. The primers used were a forward primer for high-risk HPV (5′-TGTCAAAAACCGTTGTGTCC-3′), a forward primer for low-risk HPV (5′-TGCTAATTCGGTGCTACCTG-3′), and a reverse primer (5′-GAGCTGTCGCTTAATTGCTC-3′). The reverse primer was labeled with fluorescein.

**Fig. 5:**
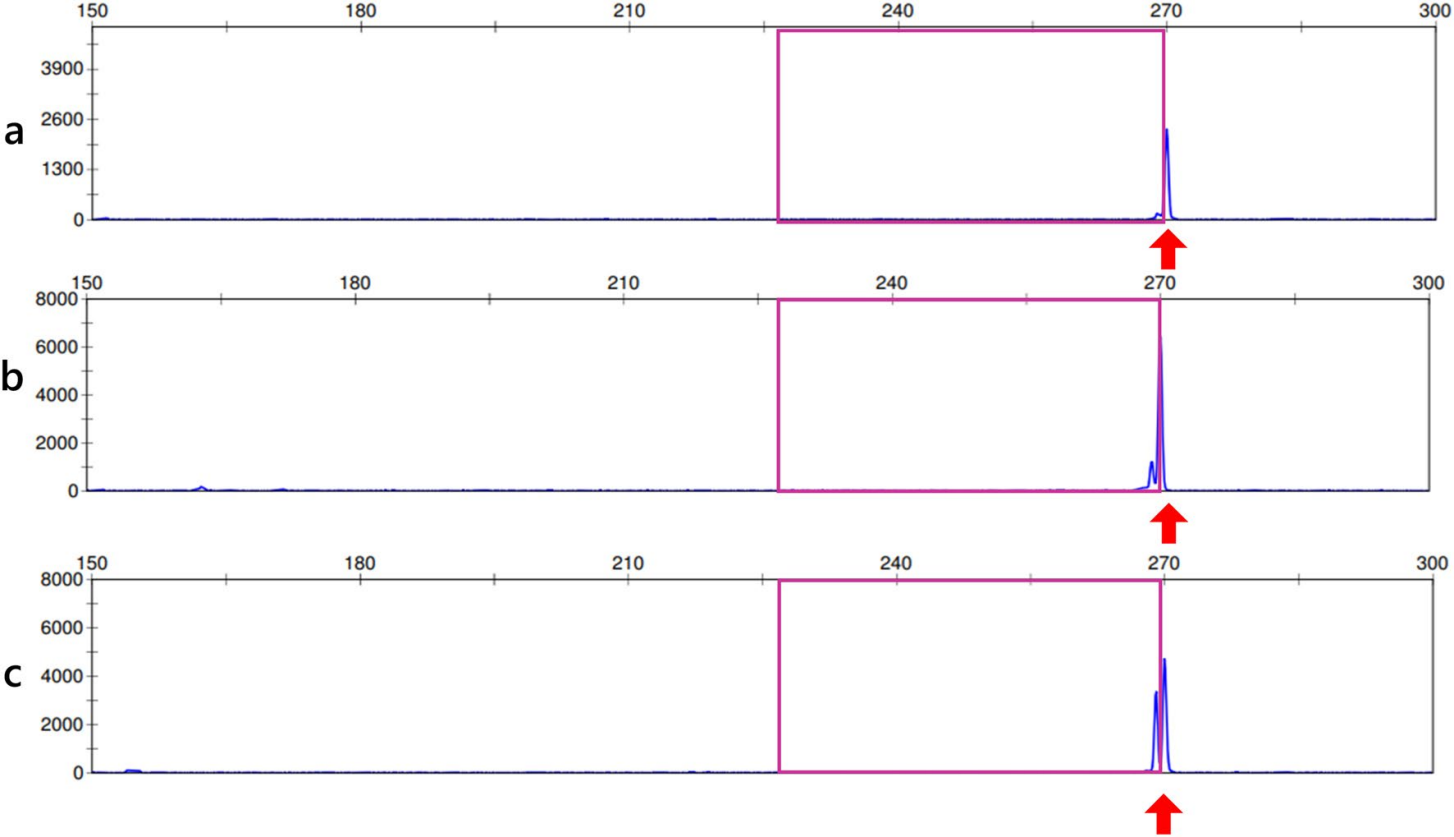
Fragment analysis (HPV) using a capillary sequencer. A single peak is visible at approximately the same position (268 bp) in all three histological tumor types (**a**: poorly differentiated adenosquamous carcinoma (glassy cell carcinoma),** b**: usual-type adenocarcinoma,** c**: squamous cell carcinoma in situ). Magenta box: amplified region (228–268 bp)

PCR products were subjected to fragment analysis using a capillary sequencer (3130xl Genetic Analyzer; Applied Biosystems, Foster City, CA, USA) and GeneMapper v4.1 (Applied Biosystems, Waltham, MA, USA). PCR positivity was defined as a single peak in the amplified (228–268 bp) region. Following incubation, a single peak was observed at 268 bp for all sampled carcinomas (Fig. 5). Table 1 shows the cytological findings, immunostaining results, and HPV genotypes of the three carcinomas.

**Table 1:** Cytological findings, immunostaining results (p16), and HPV genotypes of the three carcinomas

	PDASC (GCC)	UTAC	CIS
Cytological findings	Solid clusters Fine-to-coarse granular cytoplasm Prominent nucleoli	Papillary or tubular structures	Not recognized
Immunostaining results (p16)	+ +	+ +	+ +
HPV genotypes	HPV-18	HPV-18	HPV-18

PDASC (GCC): poorly differentiated adenosquamous carcinoma (glassy cell carcinoma), UTAC: usual type adenocarcinoma, CIS: squamous cell carcinoma in situ

+  + : diffusely positive, + : focally positive, − : negative

HPV: human papillomavirus

## Discussion

In this case, histopathological examination revealed the coexistence of poorly differentiated adenosquamous carcinoma (glassy cell carcinoma), usual-type adenocarcinoma, and squamous cell carcinoma in situ of the cervix. HPV type 18 was detected via PCR in this patient. Therefore, we performed fragment analysis to clarify the HPV infection status of the three tumors. We noted that the carcinoma regions showed identical sizes to high-risk HPV.

The main characteristic cytological findings of poorly differentiated adenosquamous carcinoma (glassy cell carcinoma) are as follows: (1) Cell clusters are characterized by a syncytial or sheet-like arrangement of cells; (2) the cytoplasm has a moderate volume and is pale and coarsely granular, i.e., with a “ground glass” appearance; (3) the nuclei are large and round with homogeneous fine-granular chromatin; and (4) a single large nucleolus and multiple small nucleoli are prominent [5, 14, 15]. In addition, although rarely, keratinization, intercellular bridges, glandular cavity formation, and accumulation of mucin in the cytoplasm may be observed [14]. The present case satisfied all four characteristics and was, therefore, considered to have presented with typical cytological findings.

Inflammatory cell infiltration of tumor cell clusters is often observed in poorly differentiated adenosquamous carcinoma (glassy cell carcinoma). Inflammatory cell infiltration of the stroma was also found in this case, along with infiltration of the tumor nests. Therefore, these histological findings were considered to be reflected in the cytological findings. Ulbright and Gersell previously described the histological features of inflammatory cell infiltration within tumor nests in glassy cell carcinoma [11]. Jung et al. [9] and Reis-Filho et al. [16] reported that cytology findings showed inflammatory cell infiltration into the clusters of glassy cell carcinomas. Therefore, inflammatory cell infiltration of cell clusters is considered an important cytological finding suggestive of poorly differentiated adenosquamous carcinoma (glassy cell carcinoma).

In the present case, a careful review of the specimen revealed a small number of clusters of papillary adenocarcinoma cells that were smaller than those of the poorly differentiated adenosquamous carcinoma (glassy cell carcinoma) and had a greater nucleus-to-cytoplasm ratio. If poorly differentiated adenosquamous carcinoma (glassy cell carcinoma) is suspected cytologically, careful screening is necessary because other malignant cells may appear.

Cervical adenocarcinoma, like squamous cell carcinoma, is frequently associated with HPV; types 16 and 18 are the most commonly detected [10]. Poorly differentiated adenosquamous carcinoma (glassy cell carcinoma) is also strongly associated with high-risk HPV, with HPV type 18 being the most common [9]. In the present case, the three carcinoma regions showed identical sizes to high-risk HPV, according to the fragment analysis. PCR results revealed that all carcinomas were HPV-18-positive. These findings are consistent with the results of p16 immunohistochemical staining. Therefore, we confirmed that all tumors, including poorly differentiated adenosquamous carcinoma (glassy cell carcinoma), were associated with HPV type 18.

A literature review revealed that cases of poorly differentiated adenosquamous carcinoma (glassy cell carcinoma) coexisting with other malignant tumors are extremely rare [11–13]. Kato et al. studied immunohistochemical staining patterns. They reported that glassy cell carcinoma showed a similar expression pattern of cytokeratin to reserve cells and immature squamous epithelial cells. They also observed squamous epithelial and glandular differentiation. Based on these findings, they stated that glassy cell carcinoma arises from pluripotent stem cells or reserve cells that can differentiate into both squamous and glandular cells [2]. Sagawa et al. reported that the frequency of coexistence of adenocarcinoma and squamous cell carcinoma in situ in the cervix is high, occurring in 8/14 (57%) patients with adenocarcinoma in situ. Based on these findings, they indicated that atypical reserve cells differentiate into both squamous cell carcinomas and adenocarcinomas [17].

In the present case, serial tumor sections were prepared and examined in detail. The squamous cell carcinoma in situ and adenocarcinoma components were very closely located to each other. However, there were no areas where the three carcinomas were clearly in contact or a transition area with an overlap. Supposing that glassy cell carcinoma and squamous cell carcinoma in situ or adenocarcinoma are of the same reserve cell origin, the possibility that these carcinomas would be seen as continuous lesions at some sites cannot be ruled out.

Based on molecular genetic analysis, previous studies have suggested that mixed-type or collision tumors in various organs are of polyclonal or monoclonal origin. Many mixed-type tumors in the uterus and lungs have been identified as having a monoclonal origin [18, 19]. In addition, Shu et al. proposed different hypotheses for the onset mechanism of collision tumors. One is that collision tumors arise from multiple clones, whereas another is that collision tumors arise from a common precursor cell [20]. Kersemaekers et al. investigated the clonality of collision tumors between cervical adenocarcinoma and cervical squamous cell carcinoma in two patients. They analyzed the loci frequently involved in cervical cancer, i.e., loss of heterozygosity (LOH) at chromosomes 1, 2, 3, 6, 11, 15, 17, 18, and X. Their study revealed partially identical LOH patterns between the adenocarcinoma and squamous cell carcinoma components of each patient and showed an additional LOH that was different in adenocarcinoma and squamous cell carcinoma. They concluded that the two tumor components initially arose from common precursor cells and displayed the same LOH pattern. Additionally, each tumor displayed a different LOH, leading to the formation of distinct tumors [21].

These findings imply that analyzing LOH patterns in tumors is extremely important when considering the mechanism of tumor development. In the present case, we needed to investigate the LOH pattern at the chromosomal loci of the three carcinomas. If all carcinomas show similar LOH patterns, it would suggests that they arose from the same clone; if they show different LOH patterns, they are likely collision tumors.

The results of the fragment analysis suggested that all carcinomas in the present case occurred owing to infection with the same high-risk HPV type. However, the clonality of these tumors has not been investigated, and further studies are needed.

## Conclusion

In this article, we report our experience with a patient in whom poorly differentiated adenosquamous carcinoma (glassy cell carcinoma) of the cervix coexisted with other malignant tumors. Poorly differentiated adenosquamous carcinoma (glassy cell carcinoma) can be diagnosed based on its characteristic cytological features. Although extremely rare, this type of carcinoma may also coexist with other malignant histological tumor types. Therefore, careful screening is required.

In this case, tumors of three different histological types coexisted. The results of the fragment analysis of the HPV isolated from the patient suggested that all the carcinomas developed following infection with the same high-risk virus type. Concerning the mechanism of tumor development in this case, further studies are needed to determine whether the tumors arose from the same clone.

## Data Availability

The data that support the findings of this study are available from the corresponding author upon reasonable request.
