# Correction: A case of coexistent poorly differentiated adenosquamous carcinoma (glassy cell carcinoma), usual-type adenocarcinoma, and squamous cell carcinoma in situ of the cervix

**DOI:** 10.1007/s00795-023-00358-9

**Published:** 2023-06-08

**Authors:** Kouki Habara, Asami Nishikori, Jin Kiyama, Manami Nakashima, Masanori Koda, Kenji Sasaki, Tomohisa Sakashita, Norifumi Tanaka, Shuji Yonehara

**Affiliations:** 1 Department of Pathology, Japan Agricultural Cooperatives Onomichi General Hospital, 1-10-23 Hirahara, Onomichi, Hiroshima, Japan; 2 Department of Molecular Hematopathology, Faculty of Health Sciences, Okayama University School of Medicine, 2-5-1 Shikata, Kita-Ku, Okayama, Japan; 3 Department of Medical Technology, Okayama University Hospital, 2-5-1 Shikata, Kita-ku, Okayama, Japan; 4 Department of Obstetrics and Gynecology, Japan Agricultural Cooperatives Onomichi General Hospital, 1-10-23 Hirahara, Onomichi, Hiroshima, Japan; 5 Department of Obstetrics and Gynecology, National Hospital Organization East Hiroshima Medical Center, 513 Jike, Saijo, Higashihiroshima, Hiroshima, Japan

**Correction: Medical Molecular Morphology** 10.1007/s00795-023-00354-z

The article “A case of coexistent poorly differentiated adenosquamous carcinoma (glassy cell carcinoma), usual-type adenocarcinoma, and squamous cell carcinoma in situ of the cervix”, written by Kouki Habara, Asami Nishikori, Jin Kiyama, Manami Nakashima, Masanori Koda, Kenji Sasaki, Tomohisa Sakashita, Norifumi Tanaka & Shuji Yonehara, was originally published electronically on the publisher’s internet portal on 02 May 2023 without open access. With the author(s)’ decision to opt for Open Choice the copyright of the article changed on 18 May 2023 to © The Author(s) 2023 and the article is forthwith distributed under a Creative Commons Attribution 4.0 International License, which permits use, sharing, adaptation, distribution and reproduction in any medium or format, as long as you give appropriate credit to the original author(s) and the source, provide a link to the Creative Commons licence, and indicate if changes were made. The images or other third party material in this article are included in the article's Creative Commons licence, unless indicated otherwise in a credit line to the material. If material is not included in the article's Creative Commons licence and your intended use is not permitted by statutory regulation or exceeds the permitted use, you will need to obtain permission directly from the copyright holder. To view a copy of this licence, visit http://creativecommons.org/licenses/by/4.0/.

